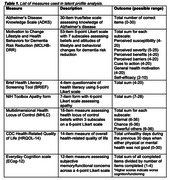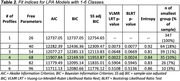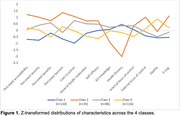# Assessing attitudes and beliefs of dementia risk and prevention in middle‐aged adults

**DOI:** 10.1002/alz.088058

**Published:** 2025-01-09

**Authors:** Stephanie M. Simone, Marina Kaplan, Tania Giovanetti

**Affiliations:** ^1^ Temple University, Philadelphia, PA USA

## Abstract

**Background:**

Healthy lifestyle behaviors (e.g., physical activity, cognitive activity) protect against cognitive decline and reduce dementia risk. Alzheimer’s disease (AD) neuropathology begins several years before the onset of clinical symptoms and disability; thus, midlife may be a critical time during which healthy lifestyle behaviors may be most effective. Motivation is essential to engage and maintain healthy lifestyle behaviors, particularly in midlife when benefits may not be observed for up to decades later. The factors that shape individuals’ motivation to engage in healthy lifestyle behaviors for dementia risk reduction remain poorly understood, but will be important for designing effective dementia prevention programs. This study identified groups of participants in midlife who possess similar beliefs and attitudes (e.g., motivation) about dementia risk and prevention using a person‐centered approach (i.e., latent profile analysis [LPA]).

**Method:**

There were 347 middle‐aged participants (M age = 54.67±5.97; 53% women; 86.5% White; M education = 14.28±2.25) from the US general population who completed an online questionnaire using Qualtrics which assessed demographics and attitudes and beliefs about dementia and lifestyle behavior changes for dementia risk reduction. Latent profile analysis was conducted using z‐transformed scores to identify subgroups of middle‐aged adults with similar beliefs and attitudes about dementia and dementia prevention (Table 1).

**Result:**

Results suggested that a four‐profile model was the best fit for the data (Table 2), yielding four distinct subgroups that differed in beliefs and attitudes about dementia and dementia prevention (Figure 1). For example, Profile 2 showed the highest level of motivation to engage in healthy behaviors and greatest concern of everyday cognition but the lowest level of AD knowledge and health literacy, whereas Profile 4 showed a variable motivation pattern but the highest levels of apathy.

**Conclusion:**

Subgroups of middle‐aged adults with different patterns of attitudes and beliefs (e.g., motivation) about dementia and dementia prevention were identified. Future work will focus on differences in healthy lifestyle behaviors (e.g., physical activity, cognitive activity) between subgroups and replication of the profiles in other cohorts. Insights gained may be used to inform who to target and how to design effective dementia prevention programs and policies for people in midlife.